# Reducing Nitrogen and Phosphorus Losses from Different Crop Types in the Water Source Area of the Danjiang River, China

**DOI:** 10.3390/ijerph16183442

**Published:** 2019-09-17

**Authors:** Mengjing Guo, Tiegang Zhang, Jing Li, Zhanbin Li, Guoce Xu, Rui Yang

**Affiliations:** 1 State Key Laboratory of Eco-hydraulics in Northwest Arid Region, Xi’an University of Technology, Xi’an 710048, China; guomengjing@xaut.edu.cn (M.G.); zhanbinli@126.com (Z.L.); xuguoce_x@163.com (G.X.); 2 Institute of Water Resources for Pastoral Area, Ministry of Water Resources, Huhhot 010020, China; 3 Inner Mongolia Water Resources and Hydropower Survey and Design Institute, Huhhot 010020, China; yaru19860129@163.com

**Keywords:** Field rainfall experiment, runoff generation, cropping patterns, N and P loss

## Abstract

Nitrogen and phosphorus are essential for plant growth and are the primary limiting nutrient elements. The loss of nitrogen and phosphorus in agricultural systems can cause the eutrophication of natural water bodies. In this paper, a field simulated rainfall experiment was conducted in a typical small watershed of the Danjiang River to study the nutrient loss process of nitrogen and phosphorus in slope croplands subjected to different crops and tillage measures. The characteristics of the runoff process and nutrient migration of different slope treatments were studied, which were the bare-land (BL, as the control), peanut monoculture (PL), corn monoculture (CL), bare land (upper slope) mixed with peanut monoculture (lower slope) (BP), corn and peanut intercropping (TCP), corn and soybean intercropping (TCS), downslope ridge cultivation (BS) slope, and straw-mulched (SC), respectively. The results showed that the runoff of CL, SC, TCS, BS, BP, PL and TCP slope types were 93%, 75%, 51%, 39%, 28%, 12%, and 6% of the those of the bare land, respectively. The total nitrogen concentration in runoff on different slope types decreased in the order of BP > PL > BS > SC > TCP > BL > CL > TCS. The BL was characterized with the highest NRL-TN (the loss of total nitrogen per unit area), with the value of 1.188 kg/hm^2^, while those of the TCP is the smallest with the value of 0.073 kg/hm^2^. The total phosphorus concentration in runoff decreasd in the order of BS > BP > PL > BL > TCP > SC > CL > TCS. The PRL-TP (the loss of total phosphorus per unit area) of BL is the largest (0.016 kg/hm^2^), while those of TCP is the smallest (0.001 kg/hm^2^). These indicate that the loss of nitrogen is much higer than that of phosphorus. The loss of nitrogen in runoff is dominated by nitrate nitrogen, which accounts for 54.4%–78.9% of TN. Slope croplands in the water source area should adopt the tillage measures of TCP and PL.These measures can reduce 85% of the runoff of nitrogen and phosphorus compared to the bare land. The results may assist in agricultural non-point source pollution control and help promote improved management of the water environment in the Danjiang River’s water source area.

## 1. Introduction

Soil erosion is one of the most severe global environmental problems. Approximately 90% of the world’s agricultural land suffers from erosion [1,2,3]. In the past 50 years, along with the expansion of agricultural developments implemented to increase crop yields, the amount of chemical fertilizer applied to cultivated land has increased continuously [4,5,6]. Nevertheless, soil erosion caused by runoff has resulted in a significant loss of nutrients, especially from sloping fields [7,8,9]. The loss of these nutrients reduces the efficiency of fertilizer and, thus, soil productivity, and leads to agricultural non-point source pollution [10,11,12]. It is reported that China’s annual soil erosion is greater than 452 million tons, and the corresponding loss of organic matter is more than twice of the total amount of fertilizers produced in this country [13]. Thus, agricultural non-point source pollution has become a serious threat to the quality of surface water, and the nutrients lost from farmland has become the dominant factor determining eutrophication of water bodies [14,15,16]. The eutrophication caused by nitrogen and phosphorus non-point source pollution has become a serious water pollution problem in many rivers in China [17]. Therefore, the losing procedure of nutrients from farmland needs to be to systematically studied to eliminate their loss and control the severity of eutrouphication in the accepting water bodies.

The present work address this problem in the area of the Danjiang River. The river is the water source of the central route project of the South-to–North Water Diversion Project, which is a very large inter-basin water transfer project in China [18,19], and by far the largest hydraulic engineering project in the world. This project aims to repair and improve the water shortage and ecological problems in Northern China, which will be completed in 2050 and have the capacity to transfer 45 billion cubic meters of water per year, equivalent to the total water volume of the Yellow River [20,21]. This project is divided into western, central, and eastern route sub-projects, which transfer water from the upper, middle, and lower reaches of the Yangtze River in Southern China, respectively [22]. The central route project will transfer 13 billion m^3^/a of water from the river to solve the water shortage problem in more than 20 large- and medium-sized cities, including Beijing, Tianjin, and Shijiazhuang, and help improve the ecological environment and agricultural water use along the related regions [23,24]. The research area of this study is concentrated in Danjiang, located in South Shaanxi Province, which is the water source area of the central route project. The water quality of this river affects the quality of the water in the region of focus directly [25]. In recent years water quality deterioration has been reported frequently due to fast development of non-point source pollution, contributed by rapid economic development that intensified agricultural activities and aggravated soil erosion in the upper reaches of the studied area [26,27,28,29]. In particular, the concentration of total nitrogen and phosphorus exceeded the Chinese Surface Water Quality Level II, and the reason would be closely associated with the agricultural non-point source pollution in the Danjiang River Basin [30,31,32]. Therefore, investigation and understanding of the process of nitrogen and phosphorus loss and their influencing factors in the water source area of the Danjiang River would be very helpful in eliminating agricultural non-point source nitrogen and phosphorus pollution. 

A number of studies have investigated the relationship between nitrogen and phosphorus loss and rainfall runoff by considering the effect of slope, soil type, and crop coverage through indoor and field rainfall experiment [33,34,35,36,37,38,39,40,41,42]. Nevertheless, the effects of different agricultural crops, tillage measures on onsite runoff, and nitrogen and phosphorus loss under field conditions have rarely been studied. The purposes of this study are to analyze the process of nitrogen and phosphorus loss for different crops (corn, peanut, and soybean) using different tillage measures, to elucidate the characteristics of nutrient loss and migration for different crops and tillage measures, and to subsequently interpret the effect of different crops and tillage measures on nutrient loss. The results could be used to control the loss of nutrients from slopes, and provide some basis for reducing the output of non-point source pollutants from the “source” and, thus, decrease the pollution load on the water environment.

## 2. Materials and Experimental Designs 

### 2.1. Study Area 

**Figure 1:**
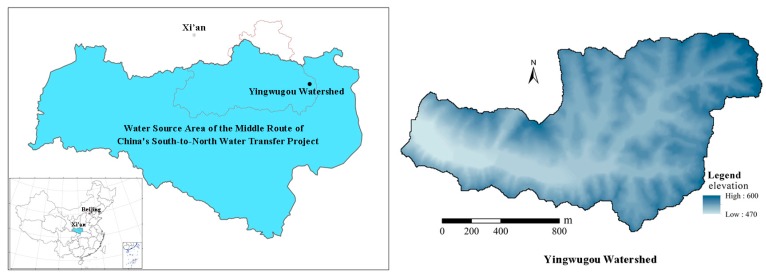
Location of the study area.

The study area is located in Yingwugou watershed in Shangnan County, Shanxi Province, China and covers an area of 1.87 km^2^, which is in the water source area of the middle route of the South-to–North Water Diversion Project (see Figure 1). The water diversion project seeks to promote Northern China’s economic growth by relaxing water constraints in a region now facing severe water shortage. The watershed was selected as: (1) it is the region with the most serious soil erosion in the whole water source area; (2) the climatic and landscape of the watershed reflect the general characteristics of this water source area; (3) the soil and slope conditions in this watershed are suitable for growing local crops, e.g., peanut, corn, and soybean. We performed the experiments under different crop types and tillage measures. The climate of the studied area is of typical Monsoon-influenced humid subtropical climate. The watershed has a mean annual temperature of 14 °C and a mean annual precipitation of 803.2 mm. The seasonal distribution of precipitation is highly uneven throughout the year with more than 60% of the annual precipitation occurs in wet seasons (from June to September). The primary landform type of the watershed is the rocky mountain and the elevation ranges from 470 m to 600 m above sea level (a.s.l.). The soil type of the watershed is dominated by yellow-brown soil and sandy loam, and the thickness of the soil layer ranges from 20–70 cm. Slope land is an important source of food and cash crops in the study watershed due to a small number of flatlands. Common food and cash crops include corn, peanuts, soybeans, and tea trees.

### 2.2. Experimental Design 

#### 2.2.1. Plot Scale Slope Type

**Figure 2:**
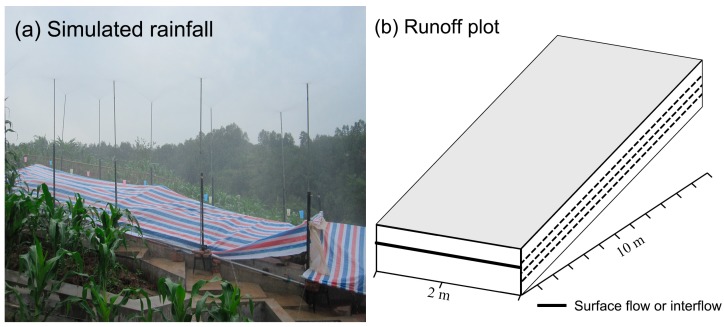
Artificial rainfall experiments on a runoff plot.

The experiments were conducted at the plot scale using the artificial rainfall simulator (see Figure 2a), and each runoff plot had similar soil and slope characteristics, with a length of 10 m, a width of 2 m and a slope of 10° (see Figure 2b).

The experiment was divided into the following two types: different crop types and different tillage measures. The former covers included peanut monoculture (PL), corn monoculture (CL), bare land (upper slope) mixed with peanut monoculture (lower slope) (BP), corn and peanut intercropping (TCP), corn and soybean intercropping (TCS), and the latter included downslope ridge cultivation (BS) slope, and straw-mulched (SC). Bare land (BL) was used as the control (Table 1). 

#### 2.2.2. Experimental design and methods 

**Figure 3:**
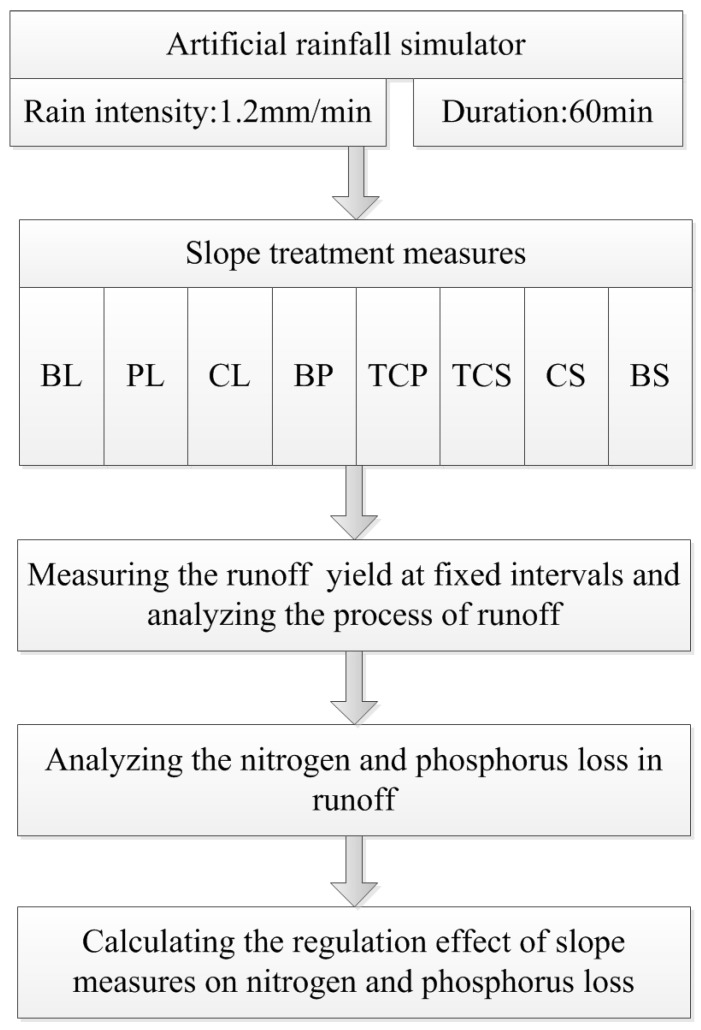
Flow chart of artificial rainfall experiments. The abbreviations in the figure denote different slope treatments, and the full name of each abbreviation is provided in Table 1.

Detailed procedures of artificial rainfall experiments are provided in Figure 3. A downward-oriented nozzle was adopted to simulate rainfall with an uniformity more than 85%. The designed rainfall intensity was 1.2 mm/min, with a duration of 60 min. These parameters were selected by taking reference from Yingwugou watershed, which is located in the middle of the Danjiang River watershed. The actual rainfall intensity was detected during the experiment. During the initial ten minutes of the simulated rainfall, the surface runoff was collected every 2 min. Afterward, the runoff of the experimental plot was collected every 5 min by using plastic buckets for measurement. The runoff flows into the plastic buckets by a collecting tank at the bottom end of the slope. Runoff nutrient indicators, including the total nitrogen (TN), nitrate nitrogen (${\mathrm{NO}}_{3}^{-}–N$), ammonium nitrogen (${\mathrm{NH}}_{4}^{+}–N$), total phosphorus (TP) and dissolved phosphorus (DP), were determined using an automatic intermittent chemical analyzer (ADA, CleverChem200, Hamburg, Hamburg state, Germany). 

The nitrogen and phosphorus losses of runoff were calculated as follows [43]:

(1) m(t)=c(t)×r(t)

where *m(t)* is the nutrient loss of the runoff at time (t), mg/min; *c(t)* is the nutrient concentration of the runoff at time (t), mg/L; *r(t)* is the amount of runoff at time (t), L/min; and t is the runoff time, min. 

The intensities of nitrogen and phosphorus losses were calculated using the following formula [36]:

(2) $$NRL=\frac{\sum_{i=1}^{n}m(t)}{A\times 100}$$

(3) $$PRL=\frac{\sum_{i=1}^{n}m(t)}{A\times 100}$$

where NRL is the loss of total nitrogen per unit area. PRL is the loss of total phosphorus per unit area. *A* is the area of the runoff plot, m^2^.

## 3. Results and Discussion

### 3.1. Runoff Process

**Figure 4:**
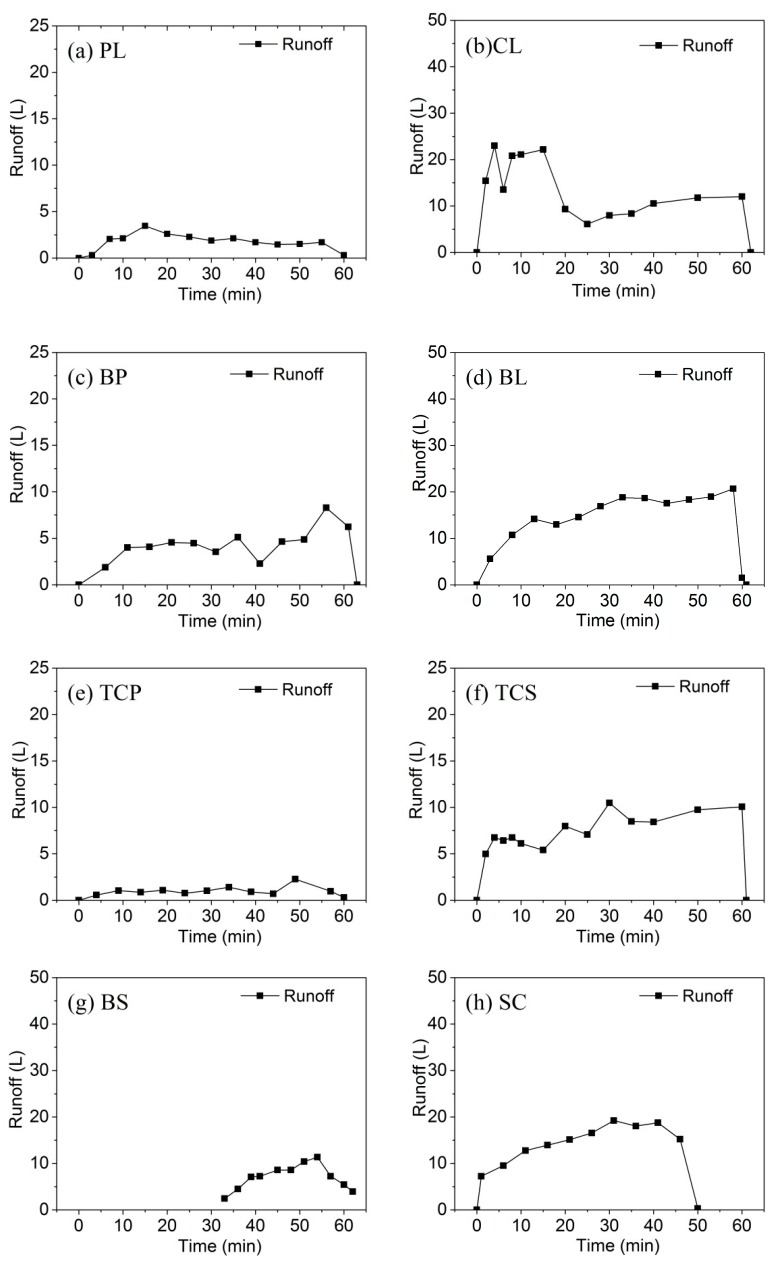
Runofft yields under different crop types.

Figure 4 presents different characteristics of the runoff procedure under different crop types. It is shown that during the early stage of a rainfall, the runoff of all the eight crop types increased with an increase in rainfall duration. The increases in the runoff rates of CL(Figure 4b), BL (Figure 4d) and SC (Figure 4f) were significantly higher than those of PL (Figure 4a) and TCP (Figure 4e). During rainfall, the runoff rates of all crops fluctuated but to different extents. After the rainfall was stopped, the runoff rates of all crops decreased rapidly, except for the case of BS. The runoff of other crops was characterized with an increase-steady-decrease trend with the increase in rainfall duration, while BS exhibited a 33-min runoff lag time, which would be due to the change in surface roughness caused by planting BS. After runoff began, BS also exhibited a runoff process that was similar to those of other crops.

The runoff of different slopes followed in the order of BL > CL > SC > TCS > BS > BP > PL > TCP and was determined to be 195.4 L, 182.1 L, 146.7 L, 98.7 L, 76.8 L, 54.9 L, 23.4 L, and 11.9 L, respectively. BL had the maximum runoff, and the runoff of CL, SC, TCS, BS, BP, PL, and TCP was 93%, 75%, 51%, 39%, 28%, 12%, and 6% of BL, respectively. The runoff of TCP and PL was 6% and 12% of BL, respectively. The crop coverage of the two slopes was greater than 80%, which indicated that high crop coverage could delay the formation of surface runoff, prolong the runoff retention time and increase water infiltration. The runoff of CL was 93% of that of BL. The similar runoff between CL and BL might be due to the following two factors: firstly, the crop spacing of corn was relatively large and the crop coverage was only 15%; secondly, the corn was at the jointing stage and the plant was small at the time of the experiment. The runoff of BP and PL was 28% and 12% of that of BL, respectively, indicating that crop coverage in the lower part of the slope was a relatively effective measure to reduce runoff.

In the intercropping of different types of crops, TCP and TCS had different effects on runoff. The runoff of TCP and TCS was 6% and 51% of that of BL, respectively, indicating that TCP was more effective in reducing runoff than TCS. In addition to higher crop coverage, the planting density of TCP was also higher than that of TCS, indicating that intercropping high and low density crops, combined with a cross planting method and appropriate crop spacing, could more effectively delay the formation of surface runoff 

The runoff of SC was also approximately that of BL, which would be due to the fact that the rainfall experiment of SC was performed after the rainfall experiment of BL. After the BL experiment, the BL surface formed a crust. Straw was then added to cover 75% of the surface, thus creating the experimental SC plot. The combination of soil crust and high straw coverage generated underlying surface conditions that were conducive to the formation of runoff, resulting in a high surface runoff of SC. 

### 3.2. Nitrogen Loss Process

#### 3.2.1. Variation of Total Nitrogen Concentration

**Figure 5:**
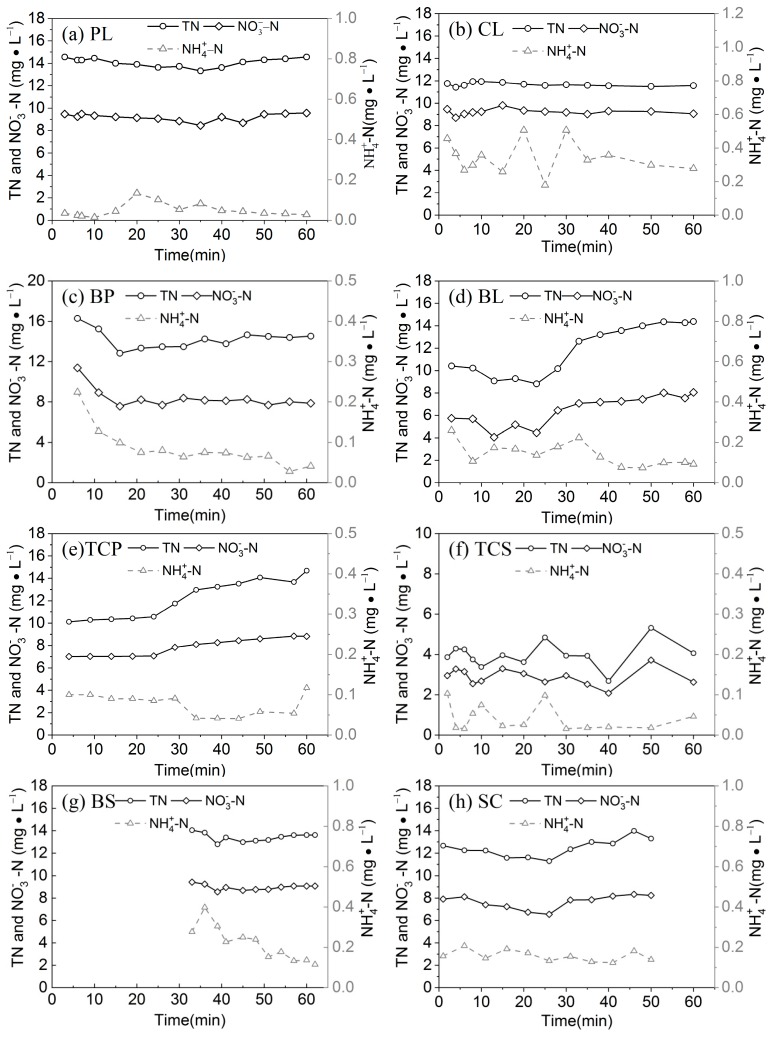
The variation of the TN, ${\mathrm{NO}}_{3}^{-}$–N, and ${\mathrm{NH}}_{4}^{+}$–N under different crop types.

The variation of the TN, ${\mathrm{NO}}_{3}^{-}$–N and ${\mathrm{NH}}_{4}^{+}$–N on different slopes are shown in Figure 5. TN concentrations on all eight slopes generally increase with an increase in the runoff duration, but with different extents. The TN concentrations of TCP and BL increased from 10.11 mg/L and 10.41 mg/L at the early stage of runoff to 14.69 mg/L and 14.38 mg/L at the end of runoff, respectively. Different slopes had different initial runoff TN concentrations and different concentration increments, indicating that the runoff conditions on different slopes had different effects on the analysis of soil nutrients. BP had the highest average runoff TN concentration of 14.23 mg/L, whereas TCS had the lowest concentration of 3.99 mg/L. The BP runoff flowed easily across BL, promoting a quick analysis of soil nutrients, whereas the peanut coverage on the lower part of the slope prolonged the runoff lag time, allowing soil nitrogen to dissolve into the runoff and leading to an increased TN concentration for BP.

#### 3.2.2. Variation of Nitrate Concentration

${\mathrm{NO}}_{3}^{-}$–N concentration in runoff exhibited a similar variation pattern as that of TN, gradually increasing with an increase in runoff duration. Crops with high coverage and intercropping prevented water flow and prolonged the contact time of runoff and soil solutes, which resulted in a higher nitrate concentration than other treatments. Runoff nitrate concentrations were also associated with the soil background content; for example, due to the low soil background values, TCS will have a low runoff nitrate concentration. 

#### 3.2.3. Variation of Ammonium Nitrogen Concentration

Unlike the TN and ${\mathrm{NO}}_{3}^{-}$–N concentrations, the ${\mathrm{NH}}_{4}^{+}$–N concentration exhibited an initial increase followed by an decrease trend with the increase in runoff duration and an overall decreasing trend. The ammonium ions of ammonium nitrogen are positively charged, allowing them to be easily absorbed onto soil particles. Therefore, the migration process of ammonium nitrogen was affected by factors such as the soil clay content and its adsorption capability of ammonium nitrogen. Furthermore, the decrease in the ammonium nitrogen concentration was much less than the TN and ${\mathrm{NO}}_{3}^{-}$–N concentrations because ammonium nitrate can be easily converted to nitrate nitrogen by soil nitrifying bacteria. 

The dissolved nitrogen in runoff primarily included nitrate nitrogen (${\mathrm{NO}}_{3}^{-}$–N) and ammonium nitrogen (${\mathrm{NH}}_{4}^{+}$–N). The average ${\mathrm{NO}}_{3}^{-}$–N concentrations were 41, 46, 49, 71, 99, 103, and 186 times higher than the average ${\mathrm{NH}}_{4}^{+}$–N concentrations for PL, TCP, BP, TCS, SC, BL, BS, and CL, respectively. The loss of ${\mathrm{NO}}_{3}^{-}$–N in runoff accounts for 54.4%–78.9% of the loss of TN in runoff (Table 2), indicating that the loss of ${\mathrm{NO}}_{3}^{-}$–N was the primary form of nitrogen loss in runoff. 

As described above, under different crop coverage and tillage measures, TN and ${\mathrm{NO}}_{3}^{-}$–N concentrations during rainfall exhibited an increasing trend, whereas the ${\mathrm{NH}}_{4}^{+}$–N concentration first increased and then decreased and was characterized with an overall decreasing trend. In the early stages of runoff, the initial concentrations of TN, ${\mathrm{NO}}_{3}^{-}$–N and ${\mathrm{NH}}_{4}^{+}$–N were only slightly different. Since different slopes had different levels of runoff, the increments of the TN, ${\mathrm{NO}}_{3}^{-}$–N, and ${\mathrm{NH}}_{4}^{+}$–N concentrations in runoff were different. The increments of the TN concentrations on TCP and BL slopes can be as much as 4 mg/L. However, TCP and BL have significantly different levels of runoff, their TN losses were 145.9 mg and 2375.0 mg, respectively, the TN loss of BL being 16.3 times that of TCP. At the end of runoff, the level of nitrogen loss of TCP and TCS continued to fluctuate, whereas those of PL, CL, BP, BL, BS and SC was relatively stable, and the TN loss was 0.163, 1.065, 0.331, 1.188, 0.512, and 0.909 kg/hm^2^, respectively. This result can be used as a export coefficient for estimating the loss of nitrogen from these slope measures at the watershed scale. 

### 3.3. The Process of Phosphorus Loss

**Figure 6:**
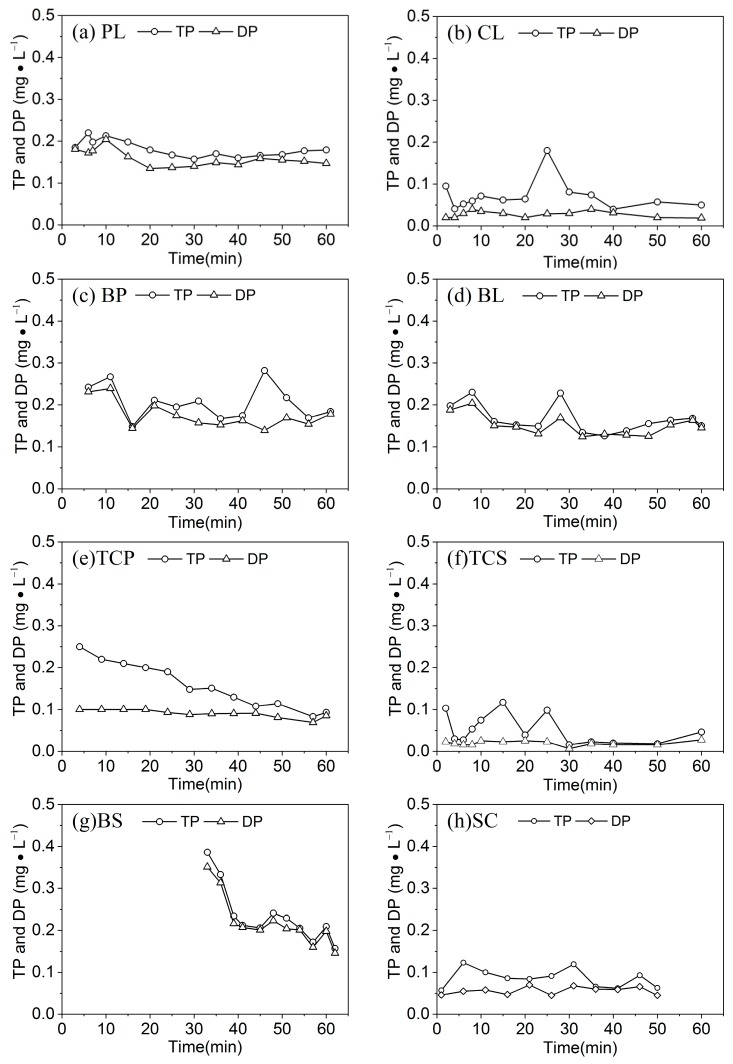
The variation of the TN and DP under different crop types.

The variation patterns of TP and DP concentrations in runoff on different slope types are depicted in Figure 6. The TP concentrations on all slopes exhibited an overall fluctuating and decreasing trend, with some localized peak concentrations. The TP concentrations on CL, BP, BL, TCP, TCS, and BS decreased from 0.09 mg/L, 0.25 mg/L, 0.20 mg/L, 0.25 mg/L, 0.10 mg/L, and 0.38 mg/L at the early stage of runoff to 0.05 mg/L, 0.18 mg/L, 0.15 mg/L, 0.09 mg/L, 0.05 mg/L, and 0.16 mg/L at the end of runoff, respectively, showing phosphorus loss with runoff is a process of reduction. Different crop covers and tillage measures resulted in different initial TP concentrations. Total phosphorous (TP) concentrations in PL and SC increased from 14.55 mg/L and 12.66 mg/L at the early stage of runoff to 14.56 mg/L and 13.29 mg/L at the end of runoff, with a 0.01 mg/L and 0.63 mg/L increment, respectively. Different levels of runoff on different slopes led to different TP concentrations. The PL had the highest TP concentration in runoff. 

The variation trend of the DP concentration in runoff was consistent with that of TP, but its change process was more gradual than that of TP. The variation trends of the TP and DP concentrations for BS and BL were approximately the same. Since BL has no crop coverage and no tillage measures, runoff can easily interact with the soil; and the TP and DP concentrations in the runoff of BL fluctuated more than those in the other slope types. 

The loss of DP accounted for 42.2%–98.3% of the loss of TP on the eight slopes (Table 3), indicating that DP was the primary form of runoff phosphorus loss, except for in the TCS and CL slope type. Intercropping and high crop coverage had a relatively strong regulation effect on phosphorus loss. SC and BP exhibited a similar amount of phosphorus loss. The intensity of TP loss decreased in the order of: BL > BS > SC > CL > BP > PL > TCS > TCP. BL had the greatest TP loss of 0.016 kg/hm^2^, whereas TCP had the least TP loss of 0.001 kg/hm^2^. This result can be used as a export coefficient for estimating the loss of phosphorus from these slope measures at watershed scale.

### 3.4. Analysis of Runoff and Nutrient Loss

A box-plot of the runoff intensity, TN and TP concentration were plotted to further analyze the association of runoff and nitrogen and phosphorus loss under different crop types and tillage measures [44]. The results showed that there were significant differences between the length of the slope (normal data), the position of the small box (mean value) and the position of the beard (anomaly) of the eight slopes, which indicated that there were significant different in runoff intensity, TN concentration and TP concentration. 

**Figure 7:**
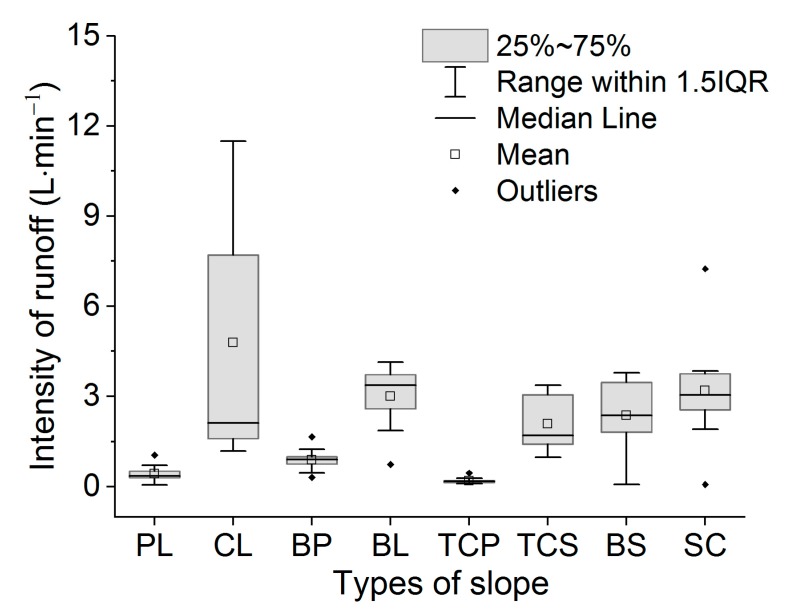
The average intensity of runoff with different slope treatments.

The average runoff intensity followed the order TCP < PL < BP < TC < BS < SC < BL < CL (Figure 7). TCP had the smallest runoff intensity of 0.2 L/min. Due to the strong fluctuation at the early stage and the end of runoff, the runoff intensity of CL was greater than BL at 4.78 L/min. From the position of the beard, the runoff of CL was shown to have the largest fluctuation range, whereas those of TCP exhibited the smallest fluctuation range. 

**Figure 8:**
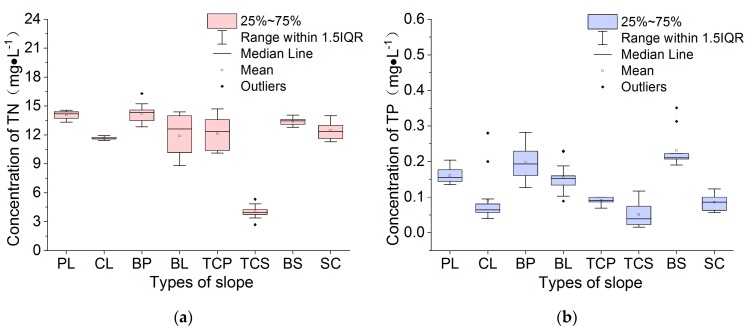
The average concentration of TN (**a**) and TP (**b**) with different slope treatments.

The average concentration of TN followed the order BP > PL > BS > SC > TC > TC > TCS (Figure 8a), and those of TP followed the order BS > BP > PL > BL > TCP > SC > CL > TCS (Figure 8b), demonstrating the obvious differences between the two concentrations. The distributions of the nutrient concentrations and the runoff intensity were also different, indicating that the nutrient concentrations were affected not only by runoff intensity but also correlated with the soil nutrient background, crop types and tillage measures. The TN and TP concentrations for TCS were the lowest of all slope types, which correlated with the low soil background value. The TN and TP concentrations for CL were the second lowest of all slope types, which correlated with the strong runoff intensity. BL and BS had exposed surfaces and high TN and TP concentrations as a result of rain splash and runoff wash. The crop type of PL was 92%, the highest level among the eight slope types. The high TN and TP concentrations of PL were due to the results fo low runoff intensity, shallow root systems, and poor nutrient retention capability. Compared with runoff intensity, the ranges in nutrient concentration levels for each slope were less, indicating that runoff can only carry a limited amount of nutrients.

### 3.5. The Regulation Effect of Slope Measures on Nitrogen and Phosphorus Loss 

Runoff was the primary driving force for the displacement and transportation of surface soil. Therefore, the key to managing soil erosion was to control runoff. If runoff can be reasonably controlled, soil erosion can be managed and limited to a minimum [45]. The effects of different crop types and tillage measures on the runoff loss of nitrogen and phosphorus were analyzed by using indicators such as runoff reduction benefit and nitrogen and phosphorus reduction benefit. The formulas for each indicator are as follows [38,46]:

(4) $$RRB=\frac{R_{b}-R_{v}}{R_{b}}\times 100\%$$

(5) $$TNRB=\frac{TR_{b}-TR_{v}}{TR_{b}}\times 100\%$$

(6) $$NNRB=\frac{NR_{b}-NR_{v}}{NR_{b}}\times 100\%$$

(7) $$ANRB=\frac{AN_{b}-AN_{v}}{AN_{b}}\times 100\%$$

(8) $$TPRB=\frac{TP_{b}-TP_{v}}{TP_{b}}\times 100\%$$

(9) $$DPRB=\frac{DP_{b}-DP_{v}}{DP_{b}}\times 100\%$$

where *R_b_* is the runoff loss of BL and *Rv* is the runoff loss under slope types. *TR_b_* is the total nitrogen loss in runoff of BL and *TRv* is total nitrogen loss in runoff under crop types or tillage measures. *RRB* is reducing runoff benefits, *TNRB* is reducing total nitrogen benefits, *NNRB* is reducing nitrate nitrogen benefits, *ANRB* is reducing ammonium nitrogen benefits, *TPRB* is reducing total phosphorus benefits, *DPRB* is reducing dissolved phosphorus benefits. The results are shown in Table 4. 

Compared with BL, the reduction rates of runoff and nitrogen and phosphorus loss of TCP were the highest (>92% for each). The reduction rates of PL were the second highest. The CL slope type had the lowest reduction rates, with ${\mathrm{NO}}_{3}^{-}$–N and ${\mathrm{NH}}_{4}^{+}$–N losses that were even greater than those of BL. According to the above analysis, the reduction rates of BS were greater than those of SC. Compared with BL, the surface area of BS increased by 20%–30%, which enlarged the rainfall area and prolonged the runoff retention time. For short rainfall durations, the runoff of BS was small which induced the small loss of nitrogen. SC had a longer runoff duration for the same duration of rainfall, and the soil nitrogen loss was large due to its high runoff. In addition, except for TCS, all types of nitrogen reduction benefits were consistent with runoff, confirming that soil nitrogen loss was dominated by runoff loss; the regulation of slope conditions regulated nitrogen loss primarily by regulating runoff. The high nitrogen reduction rate of TCS was primarily due to soybean nitrogen fixation. The experimental results showed that the nitrogen harvest index, nitrogen use efficiency and absorption efficiency of the TCS model were significantly higher than those of the soybean monoculture. 

The CL slope type had relatively large runoff levels, high runoff intensity and average nitrogen and phosphorus concentrations, which indicated a limited effect in regulating runoff and nitrogen and phosphorus loss. The crop type of the CL slope was only 15%. Furthermore, corn was a high-stem crop, which facilitated an increase in the kinetic energy of raindrops intercepted by corn leaves falling to the soil surface. Corn leaves collect rainfall to form runoff causing rill erosion. therefore, enhanced the soil erosion and baipromoted the dissolution loss of soil nutrients. The runoff may contain a amount of nutrients with the soil erosion. This leads to the runoff ${\mathrm{NO}}_{3}^{-}$–N and ${\mathrm{NH}}_{4}^{+}$–N losses of CL maybe greater than the BL which was used as a reference. Therefore, the NNRB and ANRB of CL are negative.

The reduction rates of TP and DP varied greatly among the eight slopes. Although the phosphorus loss on the slope was dominated by DP, the rate of phosphorus loss was different from that of runoff, indicating that particulate phosphorus had a particular effect on the runoff loss of phosphorus. However, under crop coverage, the amount of sediment was much smaller than that of runoff; therefore, phosphorus loss was predominantly for DP. Overall, crop types had only a slightly better regulating effect on runoff nutrient loss than from BS and SC tillage measures.

## 4. Conclusions 

In this study, the field experiment demonstrated the runoff process and the loss of nitrogen and phosphorus on eight slopes and analyzed the effects of different crops and tillage measures on nutrient loss.

(1) Based on BL, the runoff of the CL, SC, TCS, BS, BP, PL, and TCP slope types were 93%, 75%, 51%, 39%, 28%, 12%, and 6% of the those of the bare land, respectively. The results showed that the crop coverage increasing and crop interplanting can effectively regulate surface runoff and sediment yield, and the effect on sediment regulation was more obvious than that on surface runoff.

(2) The concentration of total nitrogen and nitrate nitrogen increased under different crop types and tillage measures, while the concentration of ammonium nitrogen increased first and then decreased, and the overall concentration of ammonium nitrogen decreased. Before the end of runoff, the total nitrogen, nitrate nitrogen, and ammonium nitrogen in TCP and TCS were still in the fluctuation process, while the concentrations of total nitrogen, nitrate nitrogen, and ammonium nitrogen in surface runoff of PL, CL, BP, BL, BS, and SC basically reached a stable state, in which the total nitrogen loss intensity was 0.163, 1.065, 0.383, 1.188, 0.512, and 0.909 kg/hm^2^, and the corresponding TP loss intensity was 0.002, 0.006, 0.005, 0.016, 0.009, 0.006 kg/hm^2^, respectively. The nitrogen and phosphorus output coefficient of slope farmland can be inferred from the loss intensity and the area of slope farmland.

(3) In terms of efficiency on water and sediment reduction, corn and peanuts interplanted (TCP) was the most effective for water, sediment nitrogen and phosphorus reduction (>92%), comparing with BL. The runoff and sediment reduction and nitrogen and phosphorus reduction rates of single cropping corn was the lowest. By the corn and soybean interplanting mode (TCS), the water reduction rate was 49.6% and the sand reduction rate was 73.8%, which indicated that the sand reduction efficiency was more obvious than the water. The reduction rate of nitrogen and phosphorus by TCS was also between 78.0% and 93.7%. Under straw mulch condition, the water reduction rate was 25%, but the sand reduction rate was 90.7%. Therefore, the runoff, sediment, nitrogen and phosphorus loss of slope farmland can be regulated according to the benefits of reducing water, sediment, nitrogen, and phosphorus by different crop types and tillage measures.

## Figures and Tables

**Table 1:** Design of crop types and tillage measures for artificial rainfall experiments.

Symbol	Crop or Tillage Slope Type	Rainfall Intensity	Soil Total Nitrogen	Soil Total Phosphorus	Crop Coverage	Crop Types and Tillage Measures
		(mm/min)	(g/kg)	(g/kg)	(%)	
BL	Bare land	1.20	0.72	1.11	0	Established after crop harvest
PL	Peanut	1.13	0.78	1.10	92	Crop spacing: 0.3 × 0.4 m
CL	Corn	1.07	0.77	0.83	15	Crop spacing: 1.0 × 0.5 m
BP	Bare land (upper slope) mixed with peanut (lower slope)	1.14	0.87	1.04	80	Peanuts were removed from the upper section of the slope to establish a bare land, whereas peanuts were preserved on the lower section of the slope
TCP	Corn mixed with peanut intercropping	1.14	0.87	1.14	80	Crop spacing: 0.1 × 0.5 m and (corn)
TCS	Corn mixed with soybean intercropping	1.12	0.65	0.94	55	0.2 × 0.1 m (peanut) (peanut and corn were intercropped)
BS	Downslope ridge cultivation	1.20	0.77	1.22	0	Crop spacing: 0.1 × 0.5 m (corn) and
SC	Straw-mulched bare land	1.20	0.78	0.97	75	0.3 × 0.3 m (soybean)

**Table 2:** Characteristics of nitrogen loss in runoff under different crop types.

Symbol	${NO}_{3}^{-}$–N		${NH}_{4}^{+}$–N		TN	
	Loss (mg)	Account for TN (%)	Loss (mg)	Account for TN (%)	Loss (mg)	*NRL* (kg/hm^2^)
PL	213.7	65.4	1.3	0.4	326.9	0.163
CL	1680.9	78.9	61.7	2.9	2129.2	1.065
BP	440.8	57.6	3.9	0.5	765.1	0.383
BL	1292.9	54.4	26.1	1.1	2375.0	1.188
TCP	93.8	64.3	0.8	0.6	145.9	0.073
TCS	284.1	71.8	3.7	0.9	395.9	0.198
BS	683.8	67.0	16.2	1.6	1023.6	0.512
SC	1115.5	61.3	22.9	1.3	1818.4	0.909

**Table 3:** Characteristics of phosphorus loss in runoff under different crope types.

Symbol	DP		TP	
	Loss(mg)	Account for TP (%)	Loss(mg)	PRL (kg/hm^2^)
PL	3.7	88.1	4.2	0.002
CL	5.1	42.9	11.9	0.006
BP	9.2	90.2	10.2	0.005
BL	29.6	94.6	31.3	0.016
TCP	1.1	57.9	1.9	0.001
TCS	1.9	42.2	4.5	0.002
BS	16.9	98.3	17.2	0.009
SC	8.7	67.4	12.9	0.006

**Table 4:** Relationship between cumulative runoff and sediment yield under different crop types.

Reduction Rate (%)	TCP	PL	TCS	BP	BS	SC	CL
*RRB*	93.9	88.0	49.6	72.4	60.8	25.0	6.9
*TNRB*	93.9	86.2	83.3	67.8	56.9	23.4	10.3
*NNRB*	92.7	83.5	78.0	65.9	47.1	13.7	−30.0
*ANRB*	96.8	94.9	85.8	85.0	37.8	12.3	−136.0
*TPRB*	96.6	86.5	85.6	67.5	45.2	58.8	54.3
*DPRB*	93.7	87.5	93.7	68.9	43.0	70.7	79.3
